# 

**Published:** 1892-10

**Authors:** 


### CONTENTS.

PROCEEDINGS.

The Southern Dental Association, 1892 ........ 465

National Association of Dental Faculties ........ 501

National Association of Dental Examiners ........ 505

SELECTIONS.

Creasote and its Elements ........ 509

The Indigestibility of Certain Articles ........ 510

Treatment of Neuralgia by Hypodermic Injections of Cocaine ........ 512

Hereditary Transmission of Mutilations ........ 512

Treatment of Disorders of the Teeth during Pregnancy ........ 513

EDITORIAL.

Educational ........ 514

Ohio State Dental Society—Annual Meeting ........ 516

